# Mitochondrial genome sequences illuminate maternal lineages of conservation concern in a rare carnivore

**DOI:** 10.1186/1472-6785-11-10

**Published:** 2011-04-20

**Authors:** Brian J Knaus, Richard Cronn, Aaron Liston, Kristine Pilgrim, Michael K Schwartz

**Affiliations:** 1 USDA Forest Service, Pacific Northwest Research Station, Corvallis, OR 97331, USA; 2 Department of Botany & Plant Pathology, Oregon State University, Corvallis, OR 97331, USA; 3 USDA Forest Service, Rocky Mountain Research Station, Missoula, MT 59801, USA

## Abstract

**Background:**

Science-based wildlife management relies on genetic information to infer population connectivity and identify conservation units. The most commonly used genetic marker for characterizing animal biodiversity and identifying maternal lineages is the mitochondrial genome. Mitochondrial genotyping figures prominently in conservation and management plans, with much of the attention focused on the non-coding displacement ("D") loop. We used massively parallel multiplexed sequencing to sequence complete mitochondrial genomes from 40 fishers, a threatened carnivore that possesses low mitogenomic diversity. This allowed us to test a key assumption of conservation genetics, specifically, that the D-loop accurately reflects genealogical relationships and variation of the larger mitochondrial genome.

**Results:**

Overall mitogenomic divergence in fishers is exceedingly low, with 66 segregating sites and an average pairwise distance between genomes of 0.00088 across their aligned length (16,290 bp). Estimates of variation and genealogical relationships from the displacement (*D*) loop region (299 bp) are contradicted by the complete mitochondrial genome, as well as the protein coding fraction of the mitochondrial genome. The sources of this contradiction trace primarily to the near-absence of mutations marking the D-loop region of one of the most divergent lineages, and secondarily to independent (recurrent) mutations at two nucleotide position in the D-loop amplicon.

**Conclusions:**

Our study has two important implications. First, inferred genealogical reconstructions based on the fisher D-loop region contradict inferences based on the entire mitogenome to the point that the populations of greatest conservation concern cannot be accurately resolved. Whole-genome analysis identifies Californian haplotypes from the northern-most populations as highly distinctive, with a significant excess of amino acid changes that may be indicative of molecular adaptation; D-loop sequences fail to identify this unique mitochondrial lineage. Second, the impact of recurrent mutation appears most acute in closely related haplotypes, due to the low level of evolutionary signal (unique mutations that mark lineages) relative to evolutionary noise (recurrent, shared mutation in unrelated haplotypes). For wildlife managers, this means that the populations of greatest conservation concern may be at the highest risk of being misidentified by D-loop haplotyping. This message is timely because it highlights the new opportunities for basing conservation decisions on more accurate genetic information.

## Background

Science-based management of biodiversity relies upon genetic information to identify population connectivity, conservation units, and evaluate credible divergence dates [1]. The most popular single marker for characterizing animal biodiversity is the mitochondrial genome, as mitogenetic variation tracks the matrilineal component of historical genetic diversity, migration routes [2,3] the timing of divergence events [2-5], and has relevance to fitness [6-8]. Mitochondrial haplotyping efforts typically focus on hypervariable sites within the displacement ("D") loop, since high mutation rates within this region generate substantial haplotypic variation in most species. The combination of haploidy, uniparental inheritance, and ease of genotyping this locus has led to a proliferation of conservation recommendations based partly - and in some cases entirely - on D-loop genotyping [9].

Due to the relatively small size, conserved gene content and order of animal mitochondria, intraspecific comparisons of whole mitochondrial genome variation have been possible for nearly a decade [2,3,5,10,11], although high per-sample costs limited the widespread use of such approaches in population-level studies [2,5,8]. Unlike partial genome sequencing, analysis of whole mitochondrial genomes makes it practical to partition variation into evolutionarily relevant categories (e.g., genic, proteins, synonymous, and replacement sites; putatively neutral, adaptive, and deleterious mutations), all of which can be used to produce highly accurate estimates of genealogy, divergence events, and possible adaptation to selective gradients [2,3,5].

Whole mitochondrial genome analysis also makes it possible to evaluate whether evolutionary inferences gained from subsets of the genome accurately reflect the evolutionary dynamics recorded in the full mitochondrial genome. For example, Endicott and Ho [4,12] observed dramatic differences in mutation rates, mutation saturation, and selective effects in different partitions (e.g., first, second and third codons, D-loop, rRNA) of human mitochondrial genomes; similar findings have been reported by Ingman and collaborators [13], also in humans, and by Subramanian et al. [5] in Adélie penguins (*Pygoscelis adeliae*). Using whole genome inferences, Kivisild et al. [11] proposed that portions of the mitochondrial genome have undergone positive selection during the evolution of humans. Similar information has been used to argue for adaptive divergence in specific mitochondrial genes, as shown by Castoe et al. [14] for snake evolution and Morin et al. [8] for killer whale speciation. Complete mitochondrial genome sequences can improve the resolution of maternal genealogies where subgenomic estimates are typically poorly resolved, as shown in recent studies examining the complex pattern of colonization of the New World by Native Americans [3], or the domestication history of different dog breeds [2]. The comparative stability of mitochondrial genomes over time also makes them potential targets for extracting population genomic information from paleontological specimens representing extinct [4,15-18] and their closely-related extant species. These examples implicate the mitochondrial genome as a wondrously heterogeneous marker - despite its size of only ~16 kb - for which to gain evolutionary inference.

**Figure 1:**
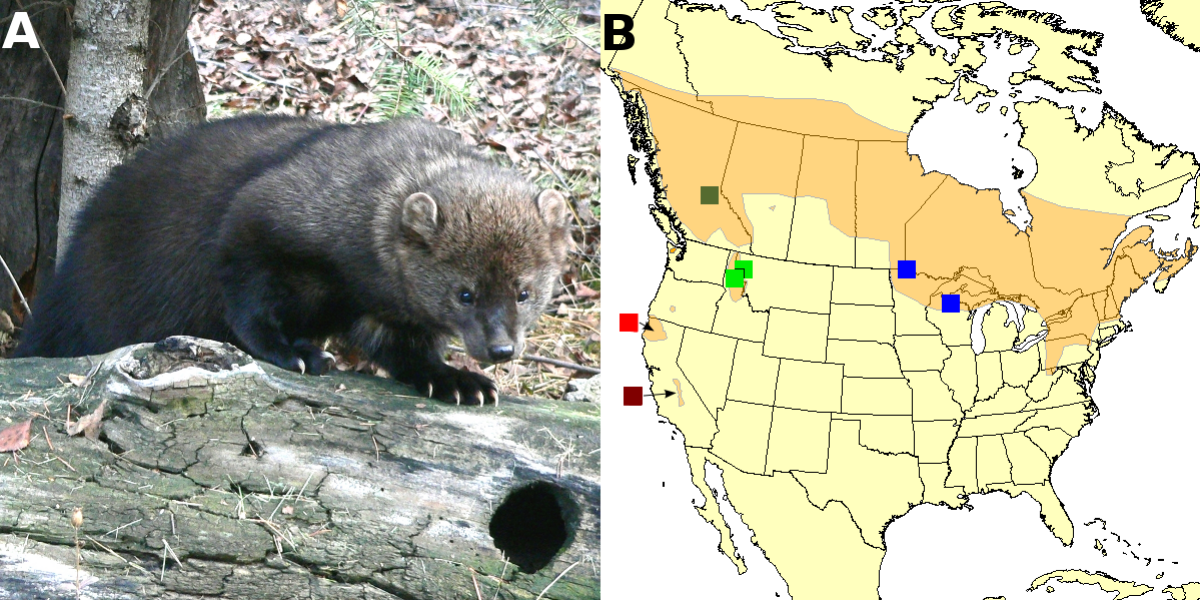
**North American fisher and its geographic distribution**. Fisher (*Martes pennanti*), a mid-sized carnivore, is distributed throughout boreal and montane North America. Subspecific classification has followed geographic subdivision of this range: ssp. *pennanti *occurs in the east (blue), ssp. *columbiana *occurs in the Northern Rocky Mountains (light and dark green), and ssp. *pacifica *is found along the Pacific coast (light and dark red).

The development of new sequencing technologies [19-23] and multiplexing approaches [24,25] now make it practical to sequence population-scale samples of small genomes at a reasonable cost, and these advancements will encourage widespread use of population-level mitogenome screening [8,15-18]. Here, we use multiplexed massively parallel sequencing to sequence and analyze complete mitochondrial genomes from fishers (*Martes pennanti*; Figure 1A), a rare carnivore in parts of its range, and one that has previously been shown to exhibit low genetic diversity in the mitochondrial [26,27] and nuclear [28,29] genomes. These data are used to evaluate the consistency of evolutionary inferences gained from partial genome genotyping (represented by D-loop sequences). We are particularly interested in evaluating: (1) how much mitochondrial genetic diversity is captured by partial genomic D-loop sequencing relative to whole genome sequencing; (2) the concordance between mitochondrial haplotypes and lineages identified with these different samples; and (3) the potential impact of mitogenome-scale information on the precision of divergence date estimates, with specific focus on differentiating divergence events (e.g., Holocene population and lineage divergence mediated via European settlement of North America) from more distant events (e.g., Pleistocene epoch or older).

The fisher is a medium sized carnivore of the mustelid family, related to marten and wolverine. In North America, where it is endemic, it has a continent-wide distribution across boreal and montane forests (Figure 1B) and is found in old, structurally-complex forests [30,31]. This species is a habitat specialist relying on snowshoe hares, red squirrels, small mammals and birds found in these forests, although it is most noted for its predation upon porcupines in some areas. Contemporary populations are thriving in eastern North America (*M. p*. ssp. *pennanti*), but the rarity and geographic isolation of Rocky Mountain (*M. p*. ssp. *columbiana*) and Pacific (*M. p*. ssp. *pacifica*) populations (Figure 1B) have resulted in petitions for listing under the U.S. Endangered Species Act, and have motivated reintroduction efforts (sometimes with non-native subspecies) across its western range [32].

Previous mtDNA genotyping based on D-loop [26] and combined D-loop and *cytochrome b *[32] sequences of fishers revealed 12 haplotypes range wide. Partitioning of these haplotypes among subspecies groupings was inconclusive. For example, some observed haplotypes were unique to geographic and taxonomic partitions. However, these authors also observed haplotypes that were shared among these partitions. One haplotype ("haplotype 1", Figure 3B; [26]) was shared among subspecies *pennanti, columbiana *and *pacifica*, and showed a geographic distribution that spanned Minnesota, Wisconsin, Montana, Idaho, British Columbia and California. In Montana and Idaho, previous mitochondrial DNA data demonstrated haplotypes present as a result of reintroductions of fishers to the Rocky Mountains from eastern and northern populations [30], and identification of a native haplotype that is hypothesized to have escaped trapping pressure and population extinction during the 20^th ^century [30]. In another case, the sharing of a haplotype among the rarest populations in the Sierra Nevada range of Southern California with a Northern California population has been used to suggest that California fisher populations were historically connected, despite a gap of 430 km in their current geographic distribution [31,32]. In both Californian and Rocky Mountain populations, management and conservation decisions have relied on matrilineal inferences estimated from partial mitochondrial genome sequences, and these data play a role in ongoing decisions regarding the status of fishers in these areas [32].

In our current analysis, we sequenced 40 complete mitochondrial genomes from fisher samples throughout their geographic range in North America, with specific emphasis on the populations of greatest conservation concern (Rocky Mountains and California; Table 1). These 40 animals represent 10 of the 12 haplotypes previously identified using the D-loop [26]. Our genome-scale analysis shows that the three subspecies of fishers do not share haplotypes, and that both Californian populations are highly distinctive from one another as well as from all other geographic regions; none of these findings are indicated by the non-coding D-loop region. These results illustrate the power that whole-genome analyses have in addressing questions of diversity and divergence at the population scale and highlight how this information can be applied to identifying evolutionary significant units to help guide conservation priorities.

**Table 1:** Sample collection localities and GenBank accession numbers.

accession	GenBank	Subspecies	Region	Collection Site	Latitude	Longitude	Previous D-Loop Designation^1^
MP1	GU121228	*pacifica*	S. California	Fresno Co, CA, USA	37.1	-119.0	1

MP2	GU121228	*pacifica*	S. California	Fresno Co, CA, USA	37.1	-119.0	1

MP3	GU121228	*pacifica*	S. California	Fresno Co, CA, USA	37.1	-119.0	1

MP4	GU121229	*pacifica*	N. California	Humboldt Co, CA, USA	41.1	-123.6	2

MP5	GU121229	*pacifica*	N. California	Humboldt Co, CA, USA	41.1	-123.6	2

MP6	GU121229	*pacifica*	N. California	Humboldt Co, CA, USA	41.1	-123.6	2

MP7	GU121230	*pacifica*	N. California	Humboldt Co, CA, USA	41.1	-123.6	1

MP9	GU121231	*columbiana*	Idaho/Montana	Idaho Co, ID, USA	46.5	-114.8	4

MP10	GU121231	*columbiana*	Idaho/Montana	Idaho Co, ID, USA	46.5	-114.8	4

MP11	GU121232	*columbiana*	British Columbia	Near Williams Lake, BC, CAN	52.1	-122.1	6

MP12	GU121232	*columbiana*	Idaho/Montana	Idaho Co, ID, USA	46.5	-114.8	6

MP13	GU121232	*columbiana*	Idaho/Montana	Ravalli Co, MT, USA	46.5	-114.3	6

MP14	GU121233	*columbiana*	Idaho/Montana	Idaho Co, ID, USA	46.5	-114.8	12

MP15	GU121233	*columbiana*	Idaho/Montana	Idaho Co, ID, USA	46.5	-114.8	12

MP16	GU121233	*columbiana*	Idaho/Montana	Mineral Co, MT, USA	47.3	-115.1	12

MP17	GU121234	*pennanti*	Great Lakes--MN	Lake of the Woods Co, MN, USA	48.7	-94.8	10

MP18	GU121235	*pennanti*	Great Lakes--MN	Lake of the Woods Co, MN, USA	48.7	-94.8	5

MP19	GU121236	*pennanti*	Great Lakes--WI	Oneida Co, WI, USA	44.5	-88.2	1

MP20	GU121236	*pennanti*	Great Lakes--WI	Oneida Co, WI, USA	44.5	-88.2	1

MP21	GU121228	*pacifica*	S. California	Fresno Co, CA, USA	37.1	-119.0	1

MP22	GU121228	*pacifica*	S. California	Fresno Co, CA, USA	37.1	-119.0	1

MP23	GU121228	*pacifica*	S. California	Fresno Co, CA, USA	37.1	-119.0	1

MP24	GU121228	*pacifica*	S. California	Fresno Co, CA, USA	37.1	-119.0	1

MP25	GU121230	*pacifica*	N. California	Humboldt Co, CA, USA	41.09	-123.6	1

MP26	GU121231	*columbiana*	British Columbia	Near Williams Lake, BC, CAN	52.1	-122.1	4

MP27	GU121231	*columbiana*	British Columbia	Near Williams Lake, BC, CAN	52.1	-122.1	4

MP28	GU121237	*columbiana*	British Columbia	Near Williams Lake, BC, CAN	52.1	-122.1	4

MP29	GU121232	*columbiana*	British Columbia	Near Williams Lake, BC, CAN	52.1	-122.1	6

MP30	GU121232	*columbiana*	British Columbia	Near Williams Lake, BC, CAN	52.1	-122.1	6

MP31	GU121232	*columbiana*	British Columbia	Near Williams Lake, BC, CAN	52.1	-122.1	6

MP32	GU121232	*columbiana*	Idaho/Montana	Idaho Co, ID, USA	46.5	-114.8	6

MP34	GU121235	*pennanti*	Great Lakes--WI	Oneida Co, WI, USA	44.5	-88.2	5

MP35	GU121235	*pennanti*	Great Lakes--WI	Oneida Co, WI, USA	44.5	-88.2	5

MP36	GU121236	*pennanti*	Great Lakes--WI	Oneida Co, WI, USA	44.5	-88.2	1

MP37	HQ705177	*columbiana*	British Columbia	Near Williams Lake, BC, CAN	52.1	-122.1	1

MP38	HQ705178	*columbiana*	British Columbia	Near Williams Lake, BC, CAN	52.1	-122.1	9

MP39	HQ705179	*columbiana*	British Columbia	Near Williams Lake, BC, CAN	52.1	-122.1	9

MP40	HQ705176	*columbiana*	British Columbia	Near Williams Lake, BC, CAN	52.1	-122.1	11

MP41	HQ705180	*columbiana*	Idaho/Montana	Idaho Co, ID	46.5	-114.8	7

MP42	HQ705180	*columbiana*	Idaho/Montana	Idaho Co, ID	46.5	-114.8	7

**^1 ^**Previous D-loop haplotype designations reflect the identifiers used for these haplotypes in previous studies [26,27,30].

## Results

### Mitogenomic variation and regional differentiation in fishers

**Figure 2:**
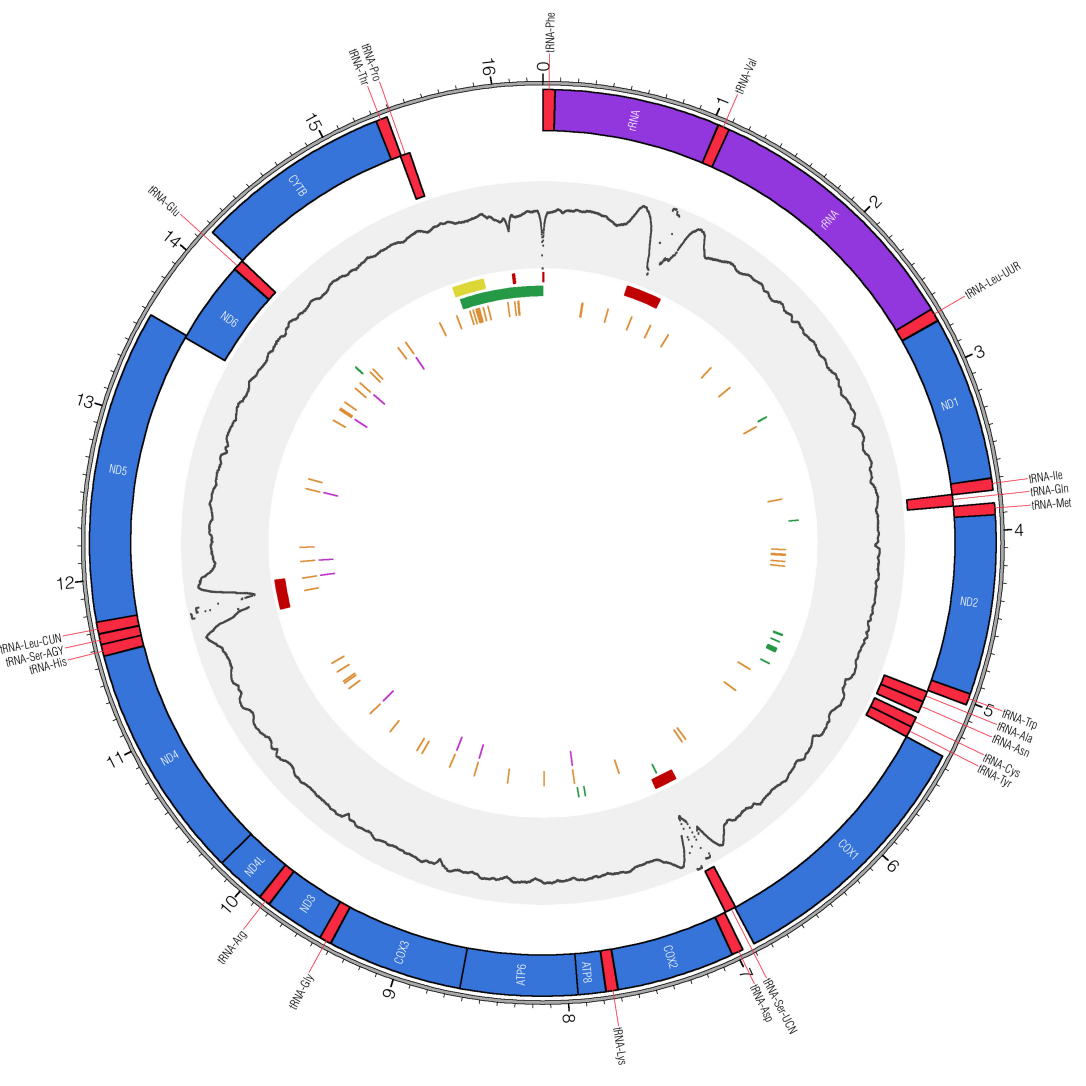
**Population variation in the fisher mitochondrial genome**. The physical organization of the fisher mitochondrial genome is shown with the position of protein coding (blue), tRNA (red), rRNA (purple) and non-coding (colorless) regions indicated. The middle grey track shows the relative sequencing depth across all 40 genomes; scale runs from 1× to 8,000× and is log transformed. Colored bars on inside track show the location of the D-loop amplicon (yellow), the non-coding portion of the mitochondrion (green), and regions that were excluded from our analysis due to insufficient read depth (red). Orange ticks represent segregating sites with magenta ticks marking amino acid substitutions.

Range-wide analysis of 40 complete fisher mitogenomes yielded an aligned data set of 16,290 bp consisting of 13 protein coding genes (11,397 bp), two ribosomal RNA genes (2,528 bp), 22 transfer RNA genes (1,515 bp), and the non-coding D-loop (299 bp)(Figure 2). Whole genome analysis revealed 15 haplotypes defined by 66 segregating sites, 19 of which are shared between two or more haplotypes, and 47 of which are found in single genomes. These variable sites combine to yield an average pairwise distance of 0.00088 in our sample of 40 genomes; averaged across samples and genomes, this equates to approximately 14.3 differences between any two mitogenomes.

Across genomes, the greatest number of nucleotide polymorphisms are located in protein coding genes (42 SNPs; 0.00369 substitutions per site), followed by the D-loop (10 SNPs; 0.03344 substitutions per site), ribosomal RNA genes (9 SNPs; 0.00356 substitutions per site) and transfer RNA genes (2 SNPs; 0.00079 substitutions per site). The exceptionally high density of variable sites in the D-loop region  - 33.4 substitutions/kb versus 3.69 substitutions/kb for the proteome - combine to reveal 10 unique haplotypes. This value is only marginally lower than the number of haplotypes revealed across all protein coding genes (n = 13), even though the proteome includes 38-times more nucleotide positions than the D-loop region.

Overall, population differentiation in mitochondrial genomes was significant among the three fisher subspecies, with 27% of the variance apportioned among our samples (*M. p. pennanti*, N = 7; *M. p. columbiana*, N = 21; *M. p. pacifica*, N = 12; AMOVA, *P *= 0.001; Table 2). A detailed examination of pairwise differentiation between populations within subspecies showed dramatic differentiation among Californian populations of fishers. Differentiation among Northern and Southern Californian fisher populations resulted in a *Φ*_PT _of 0.761 (Table 3), and the magnitude of this difference is comparable to among-subspecies differences.

**Table 2:** Analysis of molecular variance (AMOVA) for mitochondrial haplotype derived genetic distances between subspecies, between populations within subspecies, and within populations. Group membership is identified in Table 1.

Source of variation	d.f.	SS	MS	Est. Variance	%	*Φ *Statistic	Value	*P*
Among subspecies	2	0.00439	0.00220	0.00012	27%	*Φ_RT_*	0.2715	0.001

Among populations/ subspecies	3	0.00231	0.00077	0.00008	19%	*Φ_PR_*	0.2655	0.005

Within populations	24	0.00792	0.00023	0.00023	54%	*Φ_PT_*	0.4649	0.001

Total	29	0.01462	0.00320	0.00044	100%			

%, the percentage of variance explained by each sampling level. Significance of *Φ *statistics are based on 10,000 permutations of samples.

**Table 3:** Pairwise genetic differentiation in fisher mitochondrial genomes.

	*pennanti *- MN	*pennanti *- WI	*columbiana *- ID/MT	*columbiana *- BC	*pacifica *- N CA	*pacifica *- S CA
*pennanti *- MN		0.405	0.117	0.058	**0.044**	**0.017**

*pennanti *- WI	0.000		**0.012**	**0.001**	**0.001**	**0.001**

*columbiana *- ID/MT	0.227	0.313		0.111	**0.013**	**0.003**

*columbiana *- BC	0.385	0.461	0.110		**0.001**	**0.001**

*pacifica *- N CA	0.534	0.550	0.385	0.354		**0.003**

*pacifica *- S CA	0.831	0.716	0.541	0.530	0.761	

Mitochondrial DNA- based population differentiation (*Φ_PT _*below) is shown below the diagonal, and probability values estimated from 10,000 permutations are shown above diagonals. Bold indicates significant values (*P *≤ 0.05). Group membership is identified in Table 1.

### Haplotype identification and genealogical reconstructions based on complete mitochondrial genome sequences, and comparison to prior D-loop analyses

**Figure 3:**
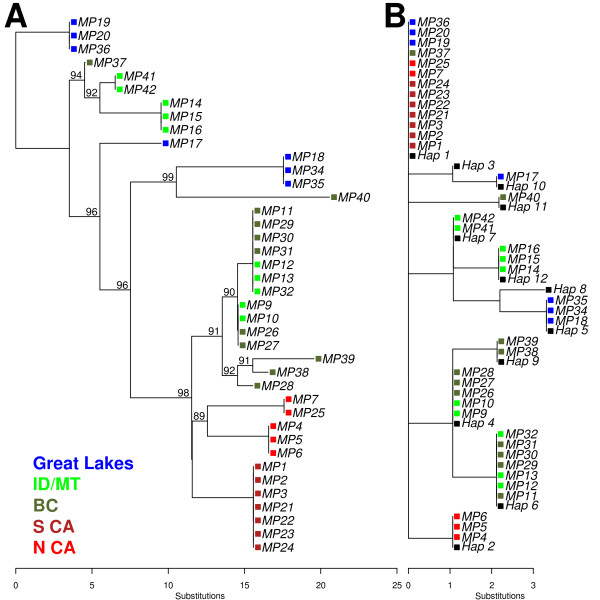
**Genealogical inferences from complete versus partial mitochondrial genomes, and the impact on haplotype identification**. Maximum likelihood trees constructed using a GTR+Γ model of nucleotide evolution: (A) complete mitochondrial genome versus (B) the D-loop region. Haplotypes are colored by geographic source. Black terminal taxa labelled "Hap 1-12" in panel 3B are D-loop haplotypes from Drew et al. [26]. Numbers above edges indicate boot strap support values > 85% derived from 1,000 replicates.

Comparisons between maximum likelihood-based evolutionary reconstructions using the complete fisher mitochondrial genome (15 haplotypes; Figure 3A) and the D-loop (10 haplotypes; Figure 3B) are of particular interest since the D-loop has previously been used to define matrilineal groups for fisher conservation (see above; [26]). Complete mitogenome sequence analysis reveals a strongly supported genealogy, with 13 of 14 possible nodes showing bootstrap support ≥ 85% (Figure 3A); this contrasts the D-loop resolution, which shows no nodal support above 85% (Figure 3B).

The genealogical estimate from complete mitochondrial genomes is complex from phylogenetic and phylogeographic perspectives, as haplotypes from the three currently designated subspecies of fishers (ssp. *pennanti*, ssp. *columbiana*, ssp. *pacifica*) show no evidence of monophyly. Similarly, haplotypes from major geographic provinces (Great Lakes region; Idaho and Montana; British Columbia; California) do not form discrete lineages, but rather a grade of closely related haplotypes (Figure 3A). The limited phylogenetic cohesiveness of mitochondrial haplotypes from different taxonomic and geographic groups appears to reflect the recency of divergence between the different geographic races of this widespread species. For example, one fisher haplotype from ssp. *pennanti *(MP18, 34 and 35, from Minnesota and Wisconsin) apparently share a more recent common ancestor with haplotypes from ssp. *columbiana *and *ssp. pacifica *than they do with other ssp. *pennanti *haplotypes (MP19, 20, and 36). Included in this grade of mitochondrial diversity are two ssp. *columbiana *haplotypes, represented by MP14-16 and MP41-42, that were previously hypothesized to represent a fisher lineage that was isolated from other Rocky Mountain lineages in ice-free refugia during Pleistocene glaciation [26]. Population level analysis of D-loop haplotype variation in this geographic region by Drew et al. [26] shows that "haplotype 7" (our MP41-42) and "haplotype 12" (our MP14-16) reach their highest frequency in the Bitterroot Mountains of western Montana/central Idaho [30], and that they are not known outside the region.

Our analysis highlights a relevant contradiction between whole genome analyses and prior analyses based on D-loop sequences. The most apparent contradiction involves the identity of the highest frequency D-loop sequence identified in prior studies, specifically "haplotype 1" [26]. This D-loop haplotype showed a nearly continent-wide distribution, being detected in populations from the Great Lakes, British Columbia, Montana, Idaho and California (Figure 3B). Whole mitogenome sequencing shows that this D-loop haplotype actually includes four distinct, non-sister lineages that sort by subspecies, and further define two geographic provenances of California (Figure 3A). Distinct haplotypes that were previously hidden within D-loop "haplotype 1" include MP19/20/36 from *M. p. ssp. pennanti *in the Great Lakes region, MP37 from *M. p. ssp. columbiana *in the Rocky Mountains of British Columbia, and *M. p. ssp. pacifica *from the Sierra (MP1-3/21-24) and the Siskiyou and Klamath (MP7/25) mountain ranges of California.

In evaluating the genetic affinities of Californian fishers, complete mitogenome sequences show much larger genetic divergence between populations in northern and southern California than has been predicted from the D-loop. Whole mitochondrial coding sequences (Figure 3A) reveal three haplotypes exclusive to Californian fishers, one that is geographically restricted to the Sierra Nevada range (S CA), and two that form a monophyletic lineage and are restricted to the Siskiyou and Klamath mountain ranges (N CA). These three haplotypes are distinctive, showing a minimum of 6 pairwise exonic differences that include several amino acid replacements (see below). In contrast, genealogical estimates from D-loop data (Figure 3B) identified two Californian haplogroups [26], including the geographically widespread, genealogically unresolved "haplotype 1" (noted above) and "haplotype 2" [26], which is equivalent to our Northern California haplotypes MP7 and MP25

We examined individual nucleotide positions that supported the competing complete mitochondrial genome and D-loop resolutions, and topological disagreement in some cases appears to be attributable to recurrent (homoplasious) mutation in variable nucleotides contained in regions typically included in D-loop genotyping (e.g., tRNA-THR plus the hypervariable region of the D-loop; table 4). An additional homoplasious mutation was identified in a genic region of the mitochondrial genome (within *cox3*; table 4). Despite the low level of mitogenomic divergence observed in our sample of fishers, recurrent mutations appear to have occurred in both the D-loop region and coding regions. When pairwise distance is exceptionally small, as is the case with Californian fishers, homoplasy in the D-loop region appears to obscure the identity and genealogical relationships recorded in the complete mitochondrial genomes.

**Table 4:** Position, polymorphism, and recurrence of mutations in the fisher mitochondrial genome.

Genomic position	423	1985	4144	5492	5768	6515	8131	8524	9166	11705	11840	12799	13722	15349	15534	15569	15576	15647	15989
**Locus**	12s rRNA	16s rRNA	ND2	COX1	COX1	COX1	ATP6	ATP6	**COX3**	tRNA Leu^CUN^	ND5	ND5	ND6	**tRNA Thr**	D-loop	**D-loop**	D-loop	D-loop	D-loop
**Locus position**	354	891	238	156	432	1179	195	588	**550**	21	95	1054	371	**44**	96	**131**	138	208	550
**Nucleotide**	A/G	A/G	C/T	A/G	C/T	C/T	A/G	C/T	**A/G**	A/G	A/G	A/G	A/G	**C/T**	C/T	**A/G**	A/G	A/G	C/T
**Amino Acid**	-	-	Leu > Leu	Gln > Gln	Asp > Asp	Phe > Phe	Gly > Gly	Leu > Leu	**Ala > Thr**	-	Asn > Ser	Ser > Gly	Ala > Val	-	-	-	-	-	-
**Changes**	1	1	1	1	1	1	1	1	**2**	1	1	1	1	**2**	1	**2**	1	1	1
**Consistency index**	1	1	1	1	1	1	1	1	**0.5**	1	1	1	1	**0.5**	1	**0.5**	1	1	1
**Homoplasy index**	0	0	0	0	0	0	0	0	**0.5**	0	0	0	0	**0.5**	0	**0.5**	0	0	0
**Retention index**	1	1	1	1	1	1	1	1	**0.6667**	1	1	1	1	**0.833**	1	**0**	1	1	1
**Rescaled ci**	1	1	1	1	1	1	1	1	**0.3333**	1	1	1	1	**0.417**	1	**0**	1	1	1

Genomic position is measured relative to the 5' end of tRNA-Phe. Locus position is relative to the first nucleotide of the start codon for coding sequences. Locus position for transfer RNAs are relative to the beginning of their 5' end. The location of the D-loop is relative to the end of tRNA-Pro, and substitutions occurring in the D-loop are indicated by bold type. Positions showing evidence of recurrent mutation are highlighted in bold print.

### Potentially non-neutral variation and the incomplete record of the D-loop

Conflation of Northern and Southern Californian mitochondrial haplotypes and their phylogenetic affinities by the D-loop (Figure 3B) is surprising given the abundance of synonymous *and *non-synonymous genomic change observed between these haplotypes. Of the 11 variable amino acid positions detected in our sample, 5 amino acid replacements are unique to northern Californian haplotypes (4 to the single haplotype represented by MP7 and MP25), accounting for a remarkable 42% of the amino acid variation in our sample of 40 individuals across North America. When the proportion of unique haplotypes for each geographic region are compared relative to the sample sizes, Californian mitogenomes (ssp. *pacifica*) show a significantly higher number of replacements than expected (41.7% versus a grand mean of 18.2%; *P *= 0.035).

**Figure 4:**
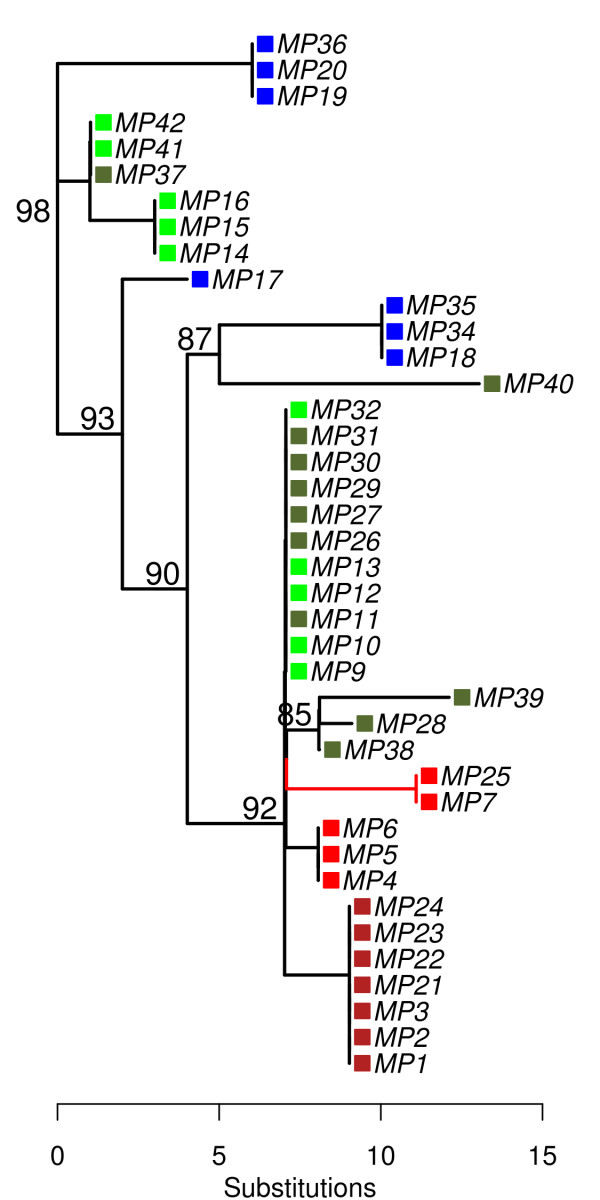
**Maximum likelihood tree for all coding nucleotides of the fisher mitochondrial genome**. The GTR+Γ model of sequence evolution was used; numbers above nodes represent bootstrap support ≥ 85. The branch colored in red indicates a significant departure from neutral evolution.

To test whether amino acid replacement rates showed evidence of non-neutral evolution, we used a codon-based genetic algorithm [33] to test whether the ratio of non-synonymous (dN) to synonymous (dS) substitutions was greater than 1. This method partitions branches of a tree (in this case, the maximum likelihood topology of the protein coding portion of the genome, with a GTR + Γ substitution model; Figure 4) into groups according to dN/dS. This analysis identified that a three rate class model had a significantly better fit than other models (see Methods). Using this model, the MP7/MP25 haplotype from Northern California was the *only *terminal that showed a probability greater than 99% of dN exceeding dS (Prob{dN > dS} = 0.999; red branch, Figure 4). Since all four substitutions on this terminal branch result in amino acid replacements, the dN/dS ratio falls in the highest rate class (0.195, 10,000) but the dN/dS ratio cannot be defined due to the absence of synonymous substitutions. This unusual substitution pattern, reflected in two independent samples (MP7, MP25), shows a clear departure from neutral evolution.

Evaluation of amino acid changes underscores two important findings. First, mitogenome sequencing shows Northern Californian haplotypes to be distinctive from each other, and from all other fisher haplogroups. At this point, we can't determine whether these changes represent an accumulation of adaptive mutations through positive selection (as has been suggested for killer whales; [8]), or the accumulation of slightly deleterious mutations through drift in small populations of asexual genomes [34]. Either way, the pattern of mutation accumulation in this lineage deviates from neutral expectations relative to our sample of haplotypes taken across North America. Irrespective of their selective relevance, these amino acid changes are uncorrelated with change in the D-loop region of the genome.

### Impact of whole genome sequencing on the precision and timing of fisher matrilineage divergence

**Figure 5:**
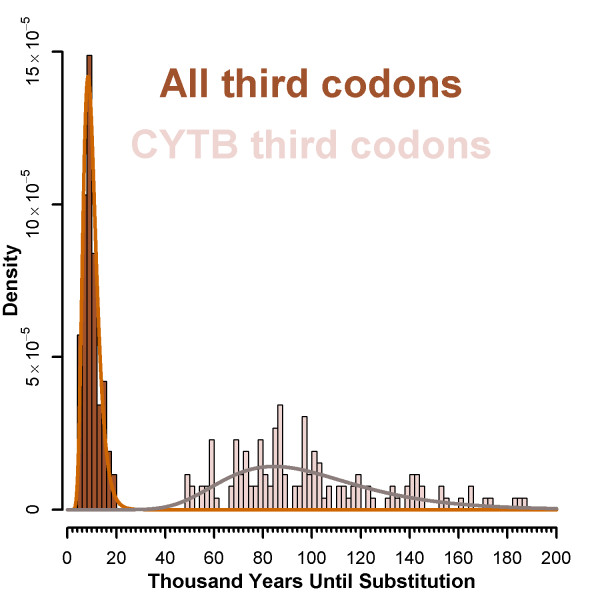
**Estimates of mutation rates and divergence dates from complete versus partial genomes**. Imposing carnivore-based estimates of mutation rates and a log-normal distribution shows that the modal time to an observed mutation for the complete fisher mitochondrial genomes is 8,428 years (95% C.I. = 5,004 - 17,364), based on all 3,796 third codon positions in the mitochondrial genome (brown). This value is significantly lower than the modal time to an observed mutation for the 379 third codons of cytochrome *b *(pink; 84,411 years, 95% C.I. = 50,115-173,914).

Our complete mitogenomes provide an opportunity to examine how whole genome sequencing might impact the accuracy of dating haplotype divergence events in closely related lineages. The use of complete mitogenomes significantly increases the precision of divergence estimates, primarily due to the increase in the number of available synonymous sites. Given the distribution of carnivore mutation rates [35] and calibrations based on *cytochrome b *(379 third codon positions), one synonymous substitution is expected in ~84 ky (50-174 ky; Figure 5). In contrast, calibrations based on the fisher mitogenome (3,799 third codon positions) instead show an expectation of one synonymous substitution every 8.4 ky (5.0 - 17.4 ky). This suggests that significant improvements in divergence date accuracy (the point estimate) and precision (decreased variance) can be obtained by simply sequencing whole organelle genomes.

This improvement in precision will be of great relevance to species showing low genetic variation and divergence, such as North American fishers. For example, haplotypes from Californian and Rocky Mountain fishers show exceedingly low pairwise divergence, averaging 1.8 synonymous substitutions per genome from their recent common ancestor (Figure 4). In light of carnivore mutation rates, these synonymous distances suggest that the most recent common mitochondrial ancestor for Northern California, Southern California, and the majority of Rocky Mountain haplotypes date to approximately 16.7 kya (9.0 - 31.3 kya). The accurate estimation of such dates clearly requires confirmation with fossils appropriate to fishers; nevertheless, this exercise shows that whole genome sequencing offers clear advantages versus partial genome sequencing with regard to the precision of recent divergence time estimates, and the ultimate perspectives they provide on the timing and origins of unique populations.

## Discussion

Our analysis highlights a relevant contradiction between whole genome analyses and prior analyses based on D-loop sequences from western fishers. Genealogical inferences based on mitochondrial D-loop variation are in conflict with the remainder of the mitogenome, and D-loop sequences underestimate the distinctiveness of the populations of greatest conservation concern due to the accumulation of independent, recurrent mutations. Results from fishers show that the mutation rate at sites within and proximal to the D-loop is sufficiently high that recurrent mutations have accumulated in a short time span; the impact of this mutational noise on genotypic identities and genealogical patterns is most pronounced in groups showing low divergence. This leads us to suggest that the fisher populations of greatest conservation concern are at the *greatest risk *of D-loop misidentification. This trend is unlikely to be limited to fishers, as low intraspecific mitochondrial divergence is widely reported in conservation genetic studies.

From a management perspective, these data are timely as fishers in California and the Rocky Mountains have been recently considered or are currently being considered for listing under the Endangered Species Act [26,32,36]. Our results confirm previous work that identifies some haplotypes from the Bitterroot Mountains of western Montana and central Idaho (e.g., MP 41-42; MP14-16) as unique relative to other known haplotypes in the U.S. Northern Rockies, British Columbia, and eastern North America. These unique mitogenomes are unlikely to represent outside reintroductions from other locations in North America, and may instead represent native haplotypes from populations that avoided early 20^th ^century extinction by persisting in Bitterroot Mountain refugia [26,30]. While additional sampling of historical and contemporary specimens will be needed to further validate this hypothesis, this haplotype group achieves its highest frequency in the Bitterroot Mountains of Montana and Idaho (Figure 3; [30]), and it is highly divergent from other Rocky Mountain fisher haplotypes. As such, these populations may warrant protection as a "distinct population segment" under the Endangered Species Act.

In California, conservation questions center around the historical versus contemporary distribution of fishers. Currently, there is a 430 km gap [31,37] between populations in Northern (the Siskiyou and Klamath ranges) and Southern (Lake Tahoe) California. Some have argued that historical fisher distributions were more or less continuous across montane regions of California, and that their current isolated distribution reflects range constriction due to anthropogenic pressure; this perspective is used to argue for reintroduction efforts that "fill the gap" between these distant geographic provenances [38]. Others have argued that fisher distributions were historically discontinuous, that migratory barriers existed prior to European settlement, and that these barriers should be preserved in contemporary fisher management plans. Key points in this argument are studies that identify fishers as a habitat specialist in the western United States, preferring low- to mid-elevation forests with diverse structure [39,40], and the absence of high-quality habitat between these populations [40].

Initial mitochondrial D-loop haplotype data by Drew et al. [26] reported a shared haplotype between Southern and Northern Californian populations, and this finding was used as evidence to argue for recent historical connectivity between these geographic provenances. This information was later contradicted by nuclear microsatellite DNA results from Wisely et al. [29], which showed large genetic divergence between Southern and Northern Californian fishers. Our results from whole mitochondrial genotyping support the findings of Wisely et al. [29] by showing high genetic divergence between Southern and Northern California fishers. Most critically, our results show that the inferences reached by Drew et al. [26] appear erroneous and are likely attributable to the unusual mutational properties of the D-loop that create (and re-create) a haplotype that mimics others ("haplotype 1") that are common across North America.

Our analysis identifies that Northern Californian haplotypes form sister lineages, and these are genealogically distinct from southern Sierra Nevada fishers. Using estimates of pairwise divergence and the synonymous mutation rate in carnivores ([26]; Figure 4), we hypothesize that the haplotypes representative of northern and southern California fishers could have diverged ~16.7 kya. This value, while based on a strict molecular clock, is consistent with previous microsatellite data [29], as well as paleontological evidence that places the earliest record of fishers in the Pacific west at < 5000 years ago [41]. If these calibrations are correct, recommendations to restore connectivity between these populations would be inconsistent with historical records [37], habitat models [40], and now contemporary molecular data.

An outstanding question in our analysis is whether contemporary fisher distributions in populations of concern primarily reflect isolation due to natural range contraction associated with the end of the Pleistocene (~10,000 ya), or disturbance associated with western settlement or 20^th ^century land management practices. Absolute divergence date estimation from molecular data at these time scales is non-trivial, as it requires precise calibration at the root of the tree (and ideally at nodes of interest) with DNA derived from sub-fossil tissues, or mutation rates calibrated to specific lineages with high quality fossils of known genealogical placement [5,42]. There is also an element of time-dependency in the use of these rates, as the average mutation rate over long evolutionary time is often significantly lower than the rate calculated from sub-fossils [5] and pedigrees [43]. Under the best circumstances, absolute divergence date estimates derived from mutation rate assumptions contain substantial and undefined error, so the dates they produce can be of unknown value when evaluating very recent divergence estimates.

Irrespective of these issues, our results show that divergence date estimates (absolute or relative) for subgenomic partitions on the order of 1/10 the size of the mitochondrial genome are highly inaccurate, and can have 95% confidence intervals measured in hundreds of thousands of years (Figure 5). The implication is that date estimates derived from small portions of mitochondrial sequence (e.g., D-loop or portions of coding genes like *cytB*) include substantial error. Improvements in the precision of estimates of genetic and relative divergence can clearly be made with whole genome sequencing, and this improved precision will be most valuable in populations showing low genetic variation and divergence, such as western fishers. It should be noted that while accurate absolute divergence dates in fishers are unlikely to be derived from distant fossil calibrations [41,44,45], late Pleistocene fisher fossils exist [41,44,45] and could be used to provide a resolution of fisher divergence dates. The growing field of paleogenomics provides striking examples of how such materials can be used to provide direct genomic information for internal calibration estimates [5].

Finally, our analysis shows that conservation genetic studies based on one or few mitochondrial gene fragments (such as those from fishers) may have sufficient power to identify ancient divergence events (e.g., Pleistocene or older), but they are certain to lack the accuracy and precision needed to confidently resolve population divergence events in the Holocene. This point has been made by others [2,5], but it is particularly relevant in the analysis of threatened, endangered, or sensitive species like fishers, where the motivating forces behind contemporary population parameters (isolation; migration; population trends) are of keen interest to conservation managers.

Our findings reinforce the need for caution when conservation and management decisions are based on small samples of the mitochondrial genome. They also raise the possibility that the incongruence between inferences from mtDNA and nuclear data sets may be at least partly attributable to the unique mutational properties of the D-loop. The ability to generate genome-scale datasets affordably means that this solution to fine-scale genealogical problems is available for conservation applications [8,23]. Wildlife managers will benefit from the more complete genomic perspectives offered by advances in genomics technologies, as population-level genetic variation has the potential to be partitioned into categories of neutral variation, putatively adaptive variation, and potentially misleading variation.

## Conclusions

○ Californian fisher populations in distinct geographic areas are represented by haplotypes that are genetically distinct from one another and from all other fisher groups. This finding is not reflected in previous research based on a small portion of the mitochondrial D-loop.

○ California populations of fisher contain at least three genetically distinct maternal lineages, and their divergence likely predates modern land management practices. One population contains a significant amount of non-neutral variation; this could be indicative of adaptive divergence or the accumulation of deleterious mutations due to small population processes.

○ Fishers in Idaho and Montana possess diverse mitogenomic lineages. One major lineage is similar to haplotypes common in British Columbia, while other lineages represented by *MP14, MP41 *represent a highly divergent, geographically restricted haplogroup.

○ These findings are broadly relevant to wildlife management, since our study shows that populations of greatest conservation concern (those showing the least genetic divergence) are at the greatest risk of being misidentified by D-loop genotyping.

## Methods

### Genome isolation, sequencing and assembly

We analyzed mtDNA from 40 fisher tissue samples collected from throughout their North American range. Total DNA was extracted using the DNeasy Tissue Kit (QIAGEN Incorporated, Hilder, Germany). Complete mitochondrial genomes were amplified in three overlapping segments using primers designed from the consensus sequence of four mustelid mitochondrial genomes (Japanese marten, *Martes malampus*, NC009678; Japanese badger, *Meles meles anakuma*, NC009677; red panda, *Ailurus fulgens*, NC009691; sea otter, *Enhydra lutris*, NC009692). Primers include: mtI-F 5'-CAAGAGGAGAYAAGTCGTAACAAG-3'; mtI-R 5'-TCTCACCTATAATTTGACTTTGACA-3'; mtII-F 5'-AAGAAAGGAAGGAATCGAACC-3'; mtII-R 5'-TTGGAGTTGCACCAATTTTTTG-3'; mtIII-F 5'-CATGGCTTTCTCAACTTTT-3'; mtIII-R 5'-CTTTGRTTTATCCAAGCACAC-3'. PCR reactions (20 μl) used ~10 ng of total genomic DNA, and were amplified using Phusion Flash polymerase (New England Biolabs). Cycling conditions included a 30 s activation at 98°C, followed by 30 cycles of 8 s at 98°C, 30 s at 59°C, and 2 min at 72°C.

Purified amplicons were pooled by individual in equimolar ratios and prepared for Illumina single-end sequencing using barcoded adapters [25]. Mitogenome pools (10 - 12 per pool) were sequenced on one lane each on an Illumina Genome Analyzer II using 40 bp microreads. Individual genomes were represented by an average of 315,000 microreads (minimum = 43,090), which is equivalent to an average of 11,340 kb of sequence per mitochondrial genome, and an average sequencing depth of 300 reads per nucleotide. The original short read sequence data is available under study number ERP000590 from the European Nucleotide Archive of the European Bioinformatics Institute http://www.ebi.ac.uk/ena/data/view/ERP000590.

Genomes were assembled using *de novo *and reference guided methods. A custom Perl script was used to sort and remove barcodes from Illumina 'qseq' files. Initial genome scaffolds were built using *de novo *assemblies (Velvet 0.7.45, [46]). BLAT 32 × 1 [47] was used to order *de novo *contigs onto the *Martes melampus *mitochondrial genome. Several rounds of reference guided assembly (RGA_blat_SNP_Q_rc4, [48]) were performed to determine whether the reference was divergent across mapped microreads, and the reference was updated after every round of assembly. Reference-guided assembly was performed until no polymorphism was detected between the reference and the microreads. MAQ [49] and BioEdit [50] were used to visualize assemblies and locate indels.

### Data analysis

Statistical analyses of DNA sequences primarily used custom R scripts [51]. Sequences and trees were manipulated using the R packages 'ape' [52], 'seqinr' [53], 'pegas' [54] and custom scripts. Maximum likelihood trees were generated using RAxML [55] at the CIPRES portal [56] and rooted with one individual from the Great Lakes that was identified as sister to our sample specimens based on phylogenies built using *Gulo gulo *(NC_009685.1), *Meles meles *(NC_011125.1), *Martes flavigulata *(NC_012141), *Martes melampus *(NC_009678) and *Martes zibellina *(NC_011579) as outgroups (not shown). In order to facilitate comparison, the D-loop was defined by the aligned sequences of Drew et al. [26] as downloaded from GenBank (299 bp). This includes a portion of tRNA-proline but was included as a representative of a D-loop amplicon as utilized in the literature. To explore how the amount of data affects statistical power of inference of divergence dates, we used estimates of species neutral evolution rate based on third codon substitutions of cytochrome *b *for 131 carnivore species [35]. Data were rescaled to reflect years until a mutation could be expected. Lognormal curves were fit to the data in R and summary statistics were derived from fitted distributions. A point estimate was made from the mode, and a 95% confidence interval was constructed from the 0.025 and 0.975 quantiles.

Analysis of molecular variance (AMOVA;[57]) was performed on DNA sequences from the three subspecies and 6 geographic populations to explore the distribution of genetic variability. For this analysis, a pairwise nucleotide distance matrix for all haplotypes was computed with MEGA4 [58], using the Kimura 2-parameter correction for multiple substitutions. This distance matrix was used as the input for AMOVA using GenAlEx ver. 6.41 [59]. In this analysis, a significant effect of subspecies (*Φ_RT_*), or populations within subspecies (*Φ_PR_*), would indicate that significant genetic structure existed at that level. *Φ_PT _*(an *F_st _*analogue for mitochondrial DNA;[57]) was used to analyze the degree of structuring among populations globally and in pairwise comparisons. Significance of the variance components was evaluated using non-parametric permutation tests with 10,000 iterations.

To test whether amino acid replacement rates were identical across genomes and lineages, we used the codon-based genetic algorithm [33] to test whether the ratio of non-synonymous (dN) to synonymous (dS) substitutions were greater than 1. This method partitions branches of a specified tree into groups according to dN/dS. This analysis identified that a three rate class model (c-AIC = 30476.6; dN/dS classes = 0.000, 0.195, 10,000) had a significantly better fit than single-rate (c-AIC = 30457.9; dN/dS = 0.177), two-rate (c-AIC = 30433.1; dN/dS classes = 0.059, 10000), or four-rate (c-AIC = 30428.1; dN/dS classes = 0.000, 0.163, 0.488, 10000) class models.

## Authors' contributions

RC, AL and MKS conceived of and designed the study. KP and RC isolated mitochondrial genomes and prepared Illumina libraries, and BJK and AL developed the pipeline for processing Illumina data. BJK constructed genome assemblies, genome alignments, and performed all sequence analyses. BJK and RC performed statistical analysis. BJK, RC, KP, AL and MKS wrote the manuscript. All authors read and approved the final manuscript.
